# The correlation of retinol-binding protein-4 and lipoprotein combine index with the prevalence and diagnosis of acute coronary syndrome

**DOI:** 10.1007/s00380-020-01627-8

**Published:** 2020-05-24

**Authors:** Yueqiao Si, Jingyi Liu, Chao Han, Ruijuan Wang, Tong Liu, Lixian Sun

**Affiliations:** Department of Cardiology, The Affiliated Hospital of Chengde Medical University, Chengde, 067000 Hebei China

**Keywords:** Acute coronary syndrome, Retinol-binding protein-4, Lipoprotein combine index, Risk factor

## Abstract

Retinol-binding protein-4 (RBP-4) along with the lipid profile plays crucial roles in Acute coronary syndrome (ACS). The study aimed to investigate the correlation of RBP-4, lipoprotein combine index (LCI), and RBP-4 + LCI with ACS. 163 ACS and 77 non-CAD in patients were consecutively enrolled in this study. The serum level of RBP-4 was measured via enzyme-linked immunosorbent assay. LCI was calculated using the formula: total cholesterol × triglyceride × low-density lipoprotein cholesterol/high-density lipoprotein cholesterol. RBP-4 ≥4 ng/ml, LCI ≥16 and LCI ≥16 + RBP-4 ≥4 ng/ml were new independent risk factors of ACS, and OR value of LCI ≥16 + RBP-4 ≥4 ng/ml was higher than that of RBP-4 and LCI combined (all *p *< 0.05). The AUC for LCI + RBP-4 was higher than that for LCI and RBP-4 individually. The risk of high LCI in 1 lesion vessel was greater than those of 2 or ≥3 lesion vessels (all *p* < 0.05). In 1 lesion vessel or ≥3 lesion vessels group, the risk associated with LCI and RBP-4 combined was higher than the risk of LCI or RBP-4 alone (all *p* < 0.05). The risk of hypertension, diabetes mellitus, smoking and history of MI increased with numbers of vessels lesion (all *p* < 0.05). Increase in RBP-4 and LCI values were found to be independent risk factors for ACS, and the risk of the combined rise in LCI and RBP-4 values was higher than LCI or RBP-4 alone. The combined tests of LCI and RBP-4 might be a potential diagnostic marker for ACS.

## Introduction

Coronary artery disease (CAD) is one of the most common causes of death worldwide, and it is responsible for more than twice the number of deaths due to cancer [1, 2]. Acute coronary syndrome (ACS) is caused due to the rupture of atherosclerotic plaques and complete or incomplete thrombosis of the coronary arteries, which is the main part of CAD [3]. Retinol-binding protein-4 (RBP-4) is a novel adipokine or hepatocytic cytokine, and its high serum levels have been associated with insulin resistance and obesity. In fact, elevated RBP-4 levels were found to be a new risk factor for cardiovascular disease [4–7]. Recently, non-traditional lipid profiles have attracted the attention of researchers, and it has been seen that the lipoprotein combine index (LCI) is higher in CAD patients than non-CAD patients. However, it is still controversial whether LCI can be established as a new risk predictor for ACS and further research is needed to clarify this [8–10]. Previous studies have reported that RBP-4 is an HDL-associated protein and it is correlated with adverse lipid profiles [11, 12]. In this study, we hypothesized that RBP-4 + LCI is a better diagnostic predictor of ACS than RBP-4 and LCI alone.

## Methods

### Participants

A total of 240 inpatients with the suspected ACS were consecutively enrolled for this study from January 2016 to May 2017 at The Affiliated Hospital of Chengde Medical College. They were divided into two groups after coronary angiography (CAG), ACS group (*n* = 163) and non-CAD group (*n* = 77). The Inclusion Criteria for ACS group included age ≥18 years; unstable angina; non-ST elevation myocardial infarction; and ST-elevation myocardial infarction [13]; CAG: the stenosis at least or more than 50% in one or more of the left main, left anterior descending, left circumflex, right coronary, or their main branches. The Inclusion Criteria for the non-CAD group included age ≥18 years; suspected ACS without any stenosis of coronary arteries or stenosis less than 50% of the left main, left anterior descending, left circumflex, right coronary, or their main branches. The Exclusion Criteria for both ACS group and non-CAD group included infectious diseases, malignant tumors, immune system diseases, connective tissue disease, severe valvular heart disease, aortic dissection, constrictive pericarditis, hypertrophic cardiomyopathy and pregnancy. This study was carried out in accordance with the World Medical Association’s Code of Ethics (Helsinki Declaration), and approved by the Institutional Review Boards of The Affiliated Hospital of Chengde Medical University. All subjects provided written informed consent.

### Demographic and clinical data

The baseline characteristics of the demographic and clinical data included the age, gender, height and weight, systolic blood pressure, diastolic blood pressure, the circulation level of total cholesterol (TC), triglyceride (TG), low-density lipoprotein cholesterol (LDL-C), and high-density lipoprotein cholesterol (HDL-C). The risk factors for ACS such as diabetes, hypertension, dyslipidemia and ischemic stroke were carefully noted. Following this, the LCI [10] was calculated using the formula, TC × TG × LDL-C/HDL-C.

### Enzyme-linked immunosorbent assay

Peripheral blood samples of all the subjects were taken from the antecubital vein after overnight fasting and prior to CAG. The blood samples were allowed to stand for 30 min and centrifuged at 5000*g* for 10 min. Then, the serum was aliquoted and stored at − 80 °C until further use. The serum level of RBP-4 was measured using an enzyme-linked immunosorbent assay (ELISA) kit.

### Statistical analysis

Statistical analyses were performed using Statistical Package for Social Sciences, version 19 (SPSS Inc., Chicago, IL, USA) software. Kolmogorov–Smirnov test was used to analyze all continuous data with normal or skewed distribution. Mean ± standard deviation and quartile M (QR) were used to express normal and skewed continuous data, respectively. The categorical data were expressed as percentages. Differences in the patient characteristics between the ACS group and non-CAD group were tested with Mann–Whitney *U* test for continuous variables and Chi square testing was used for categorical variables. The receiver operation characteristic (ROC) curve was used to determine the best diagnostic cut-off point for RBP-4 and LCI, as well as the AUC of RBP-4, LCI and RBP-4 + LCI. All the categorical variables were analyzed using univariate logistic regression analysis, then, variables with *p* < 0.05 obtained from the univariate analysis were included in the multivariate logistic regression analysis and multinomial logistic regression analysis. Multivariate logistic regression model 1 and 2 were established by adjusted RBP-4, LCI, and RBP-4 + LCI. Multinomial logistic regression analysis model was established based on 4 groups according to the number of lesion vessels, and then, the RBP-4, LCI and RBP-4 + LCI values were adjusted to establish the models 1, 2 and 3. The level of significance for all the analyses was set at *p* < 0.05.

## Results

### Baseline characteristics of the ACS group and non-CAD group

The mean age and BMI as well as the number of subjects with smoking history were significantly higher in the ACS group than in the non-CAD group (all *p* < 0.05). Chest pain and abnormal wall motion were more common in the ACS group than non-CAD group (both *p* < 0.05). Compared to the non-CAD group, the morbidity due to myocardial infarction, dyslipidemia, hypertension, and diabetes mellitus were significantly higher in the ACS group (all *p* < 0.05). The serum lipid profiles of the patients in the ACS group showed significantly higher TC, TG and LDL-C levels than those in the non-CAD group (all *p* < 0.05). Yet, there was no significant difference between the two groups in terms of the HDL-C level and the prevalence of ischemic stroke (both *p* > 0.05). In the ACS group, the prevalence of RBP-4 ≥4 ng/ml, LCI ≥16, RBP-4 ≥4 ng/ml + LCI ≥16 were significantly higher than non-CAD group (all *p* < 0.05). Moreover, the number of patients with 1, 2 and ≥3 lesion vessels in the ACS group were 53, 48 and 62, respectively (Table 1).

**Table 1:** Baseline characteristics of the ACS group and non-CAD group

Variables	ACS group (*n* = 163)	Non-CAD group (*n* = 77)	*χ*^2^/*Z*	*p*
Male	120 (73.6)	49 (63.6)	1.595	0.114
Age (years)	60 ± 10	57 ± 10	2.223	0.027
BMI (kg/m^2^)	25.5 ± 2.4	24.6 ± 2.4	2.343	0.020
Smoking (%)	99 (60.7)	28 (36.4)	2.707	< 0.001
Family history of CAD (%)	38 (23.3)	14 (18.2)	1.368	0.368
History of MI (%)	51 (31.3)	3 (3.9)	11.232	< 0.001
Abnormal heart wall motion (%)	93 (59.2)	19 (26.8)	3.977	< 0.001
Chest pain (%)	138 (84.7)	20 (26.0)	15.732	< 0.001
Dyslipidemia (%)	74 (45.4)	15 (19.5)	3.437	< 0.001
Hypertension (%)	111 (68.1)	40 (51.9)	1.975	0.016
Diabetes mellitus (%)	64 (39.3)	15 (19.5)	2.672	0.002
Ischemic stroke (%)	33 (20.2)	14 (18.2)	1.142	0.707
Systolic blood pressure (mmHg)	136 (123,160)	136 (121,145)	− 1.619	0.105
Diastolic blood pressure (mmHg)	81 (75,90)	80 (75,90)	− 0.057	0.955
TC (mmol/l)	4.2 ± 1.0	3.9 ± 0.9	2.009	0.046
TG (mmol/l)	1.7 (1.2,2.5)	1.4 (1.1,2.1)	− 2.007	0.038
LDL-C (mmol/l)	2.1 ± 0.9	1.9 ± 0.8	2.231	0.027
HDL-C (mmol/l)	1.0 (0.9,1.2)	1.1 (0.9,1.3)	− 1.125	0.260
Coronary angiography				
1 vessel (%)	53 (32.5)	–	–	–
2 vessels (%)	48 (29.4)	–	–	–
≥3 vessels (%)	602 (36.8)	–	–	–
RBP-4 ≥4 ng/ml (%)	136 (83.4)	33 (42.9)	6.716	< 0.001
LCI ≥16 (%)	78 (47.9)	19 (24.7)	2.801	0.001
RBP-4 ≥4 ng/ml + LCI ≥16 (%)	69 (42.3)	10 (13.0)	4.918	< 0.001

Data presented as number (%) of patients, median (interquartile range)

*BMI* body mass index, *MI* myocardial infarction, *TC* total cholesterol, *TG* triglyceride, *LDL*-*C* low-density lipoprotein cholesterol, *HDL*-*C* high-density lipoprotein cholesterol, *RBP*-*4* retinol-binding protein 4

**Fig. 1:**
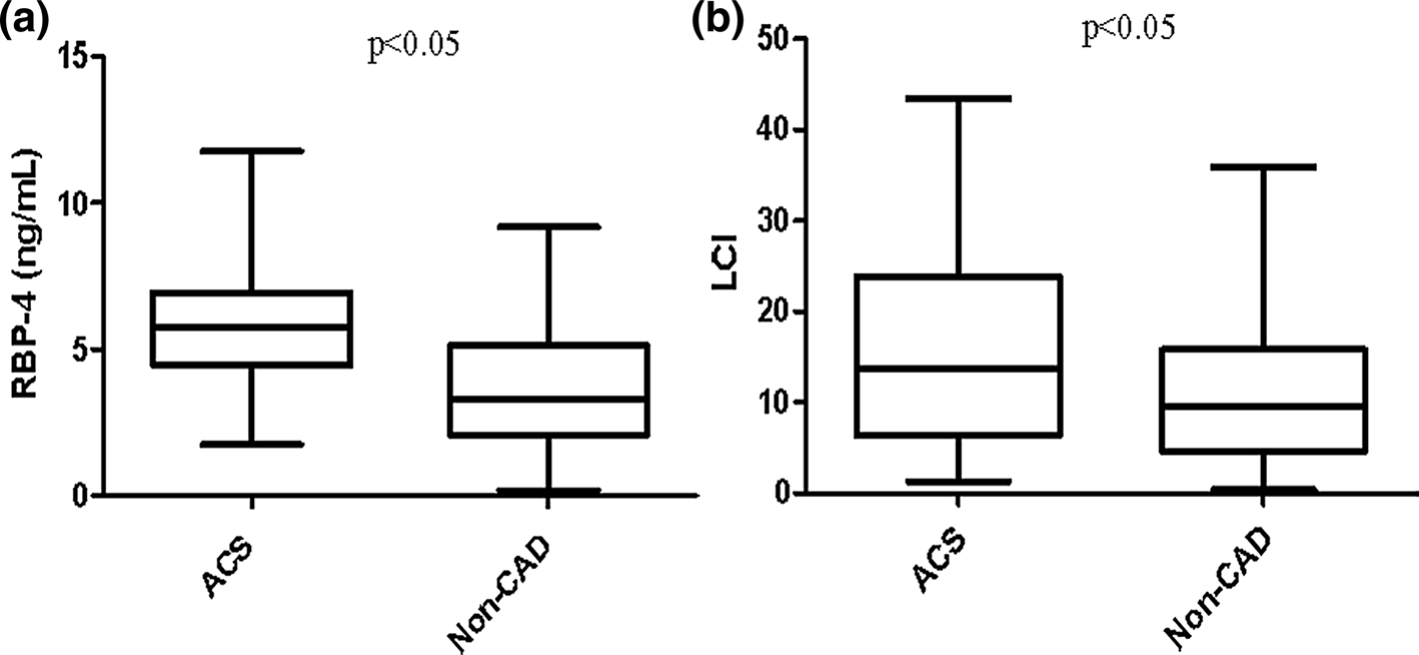
**a** The level of RBP-4 in ACS group and Non-CAD group, **b** the value of LCI in the ACS group and non-CAD group

**Fig. 2:**
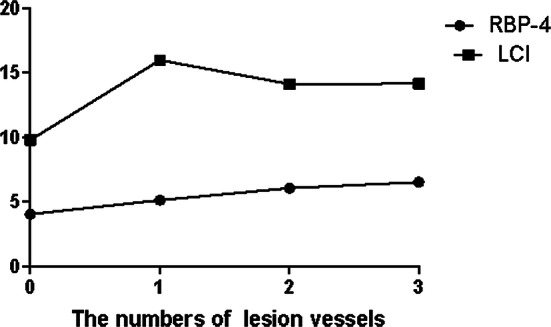
The level of RBP-4 and the value of LCI among the lesion vessels group with varying lesion numbers

The RBP-4 levels and the LCI values were significantly higher in the ACS group than the non-CAD group (Fig. 1). Compared to the 4 groups with different numbers of lesion vessels, the RBP-4 level increased with an increase in the number of lesion vessels. The LCI value was significantly higher in patients with lesion vessels than patients without lesion vessels, with the highest being in the group with 1 lesion vessel (Fig. 2).

### Receiver-operating characteristic curve analyses of ACS group and non-CAD group subjects

**Fig. 3:**
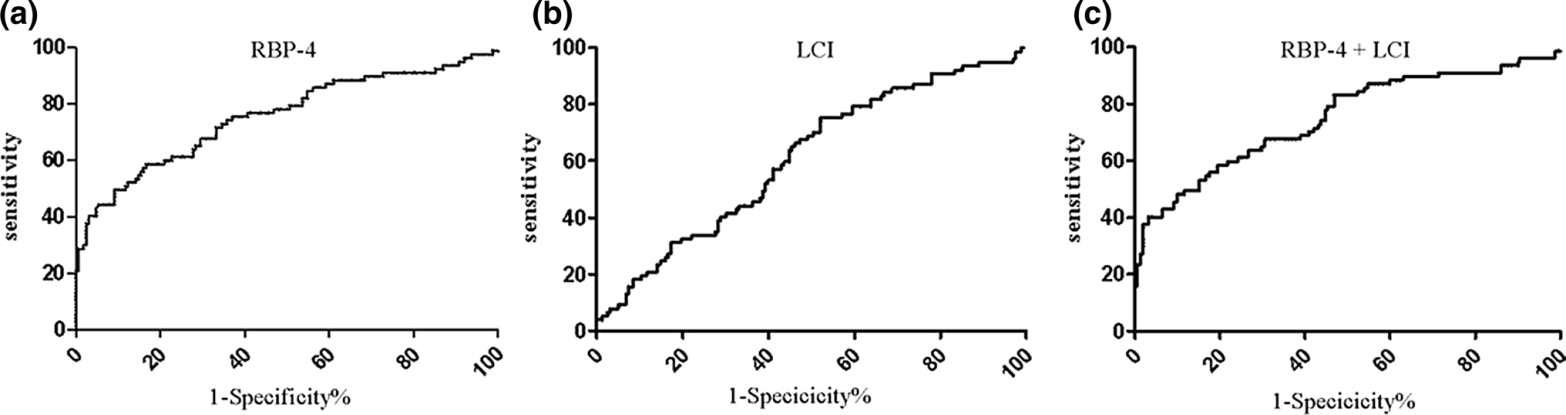
**a** Receiver operation characteristic (ROC) curve of RBP-4, **b** ROC curve of LCI, and **c** ROC curve of RBP4+LCI

The AUC of the RBP-4 was 0.754 (95% CI 0.638–0.825, *p* < 0.001). The optimal diagnostic cut off point for RBP-4 was 4 ng/ml. The sensitivity and the specificity were 84 and 57%, respectively. The AUC of the LCI was 0.608 (95% CI 0.532–0.683, *p* = 0.007). The optimal diagnostic cut off point for LCI was 16 ng/ml, and the sensitivity and the specificity were 48 and 75%, respectively. The AUC of the LCI + RBP-4 was 0.761 (95% CI 0.691–0.830, *p* < 0.001), and the sensitivity and the specificity were 83 and 58%, respectively. Surprisingly, the AUC of the LCI + RBP-4 was higher than the AUC of the LCI and RBP-4 (Table 2; Fig. 3).

**Table 2:** ROC curve analyses of the ACS group and non-CAD group

Variable	AUC	SE	*p*	95% CI	Se (%)	Sp (%)	Cut off
RBP-4	0.754	0.036	< 0.001	0.638–0.825	84	57	4
LCI	0.608	0.038	0.007	0.532–0.683	48	75	16
LCI + RBP-4	0.761	0.036	< 0.001	0.691–0.830	83	58	–

*AUC* area under curve, *RBP*-*4* retinol-binding protein 4, *LCI* lipoprotein combine index

### Multiple logistic regression of ACS risk factors

Multivariate logistic regression model 1 analysis showed that hypertension, diabetes mellitus, smoking, history of MI, RBP-4 ≥4 ng/ml and LCI ≥16 were independent risk factors of ACS, and the odds ratio (OR) of these factors were 2.284 (1.123, 4.644), 2.716 (1.251, 5.899), 4.082 (1.996, 8.345), 10.102 (2.803, 36.404), 4.435 (2.202, 8.931) and 2.496 (1.209, 5.154), respectively (all *p* < 0.05). Multivariate logistic regression model 2 analysis showed that hypertension, diabetes mellitus, smoking, history of MI and LCI ≥16 + RBP-4 ≥4 ng/ml were independent risk factors of ACS, the odds ratio (OR) of these factors were 2.546 (1.282, 5.054), 3.185 (1.490, 6.808), 4.076 (2.043, 8.130), 12.305 (3.514, 43.087) and 5.299 (2.348, 11.957), respectively (all *p* < 0.05). The OR value of the history of MI was the highest among those risk factors in the two models. RBP-4 ≥4 ng/ml, LCI ≥16 and LCI ≥16 + RBP-4 ≥4 ng/ml were all new independent risk factors of ACS. In addition, the OR value of LCI ≥16 + RBP-4 ≥4 ng/ml was higher than the OR value of RBP-4 ≥4 ng/ml and LCI ≥16. (Table 3).

**Table 3:** Multiple logistic regression of ACS risk factors

Variable	Model 1			Model 2		
	OR	95% CI	*p*	OR	95% CI	*p*
Hypertension	2.284	1.123–4.644	0.023	2.546	1.282–5.054	0.008
Diabetes mellitus	2.716	1.251–5.899	0.012	3.185	1.490–6.808	0.003
Smoking	4.082	1.996–8.345	< 0.001	4.076	2.043–8.130	< 0.001
History of MI	10.102	2.803–36.404	< 0.001	12.305	3.514–43.087	< 0.001
RBP-4 ≥4 ng/ml	4.435	2.202–8.931	< 0.001	–	–	< 0.001
LCI ≥16	2.496	1.209–5.154	0.013	–	–	0.013
LCI ≥16 + RBP-4 ≥4 ng/ml	–	–	–	5.299	2.348–11.957	< 0.001

*MI* myocardial infarction, *RBP*-*4* retino-binding protein 4, *LCI* lipoprotein combine index

### Multinomial logistic regression model for risk factors of different number of lesion vessels

To investigate the strength of the association between different lesion vessels and the factors such as RBP-4, LCI, and RBP-4 + LCI, we established multinomial logistic regression analysis model 1, 2, 3 with adjusted RBP-4, LCI and RBP-4 + LCI, respectively. Smoking, history of MI, RBP-4 ≥4 ng/ml, LCI ≥16 and RBP-4 + LCI were found to be independent risk factors of 1 lesion vessel; hypertension, diabetes mellitus, smoking, history of MI, RBP-4 ≥4 ng/ml, LCI ≥16 and RBP-4 + LCI were independent risk factors of 2 lesion vessels; and finally, hypertension, diabetes mellitus, smoking, history of MI, RBP-4 ≥4 ng/ml, LCI ≥16 and RBP-4 + LCI were independent risk factors of 3 lesion vessels (all *p* < 0.05).

In the 1 lesion vessel group and ≥3 lesion vessels group, the odds ratio (OR) of LCI ≥16 + RBP-4 ≥4 ng/ml were higher than the odds ratio (OR) of LCI ≥16 or RBP-4 ≥4 ng/ml alone. However, in 2 lesion vessels group, the odds ratio (OR) of RBP-4 ≥4 ng/ml was higher than the odds ratio (OR) of LCI ≥16 + RBP-4 ≥4 ng/ml and LCI ≥16. In addition, multinomial logistic regression models 1, 2, 3 for risk factors of 1, 2, 3 lesion vessels group showed that the odds ratio (OR) of hypertension, diabetes mellitus, smoking and history of MI increased with the increase in the number of causing lesion vessels (Table 4).

**Table 4:** Multinomial logistic regression model for risk factors of different number of vascular lesions

Variable		1 lesion vessel			2 lesion vessels			≥3 lesion vessels		
		OR	95% CI	*p*	OR	95% CI	*p*	OR	95% CI	*p*
Model 1	Hypertension	1.153	0.529–2.513	0.721	2.442	1.009–5.907	0.048	2.876	1.214–6.816	0.016
	Diabetes mellitus	2.067	0.848–5.034	0.110	3.936	1.594–9.717	0.003	4.718	1.969–11.306	0.001
	Smoking	3.323	1.498–7.373	0.003	3.390	1.438–7.995	0.005	3.503	1.528–8.027	0.003
	History of MI	4.559	1.325–15.691	0.016	6.068	1.728–21.310	0.005	9.876	2.946–33.116	< 0.001
	RBP-4 ≥4 ng/ml	3.643	1.597–8.309	0.002	6.587	2.385–18.197	< 0.001	4.749	1.902–11.859	0.001
Model 2	Hypertension	1.384	0.638–3.002	0.411	2.983	1.256–7.084	0.013	3.453	1.475–8.082	0.004
	Diabetes mellitus	1.862	0.756–4.584	0.177	3.711	1.517–9.077	0.004	4.448	1.861–10.635	0.001
	Smoking	3.434	1.552–7.601	0.002	3.458	1.498–7.893	0.004	3.584	1.586–8.097	0.002
	History of MI	6.628	1.952–22.505	0.002	9.355	2.724–32.130	< 0.001	14.575	4.417–48.092	< 0.001
	LCI ≥16	3.627	1.637–8.036	0.002	2.993	1.292–6.934	0.011	2.884	1.269–6.554	0.011
Model 3	Hypertension	1.360	0.621–2.978	0.128	2.954	1.230–7.092	0.015	3.413	1.445–8.062	0.005
	Diabetes mellitus	2.020	0.818–4.990	0.128	3.887	1.579–9.572	0.003	4.672	1.944–11.244	0.001
	Smoking	3.516	1.573–7.859	0.002	3.565	1.526–8.326	0.003	3.679	1.613–8.393	0.002
	History of MI	6.182	1.811–21.107	0.004	8.865	2.559–30.707	0.001	13.847	4.168–46.003	< 0.001
	LCI + RBP-4	5.863	2.308–13.991	< 0.001	5.494	2.140–14.105	< 0.001	4.996	1.974–12.641	0.001

*MI* myocardial infarction, *RBP*-*4* retinol-binding protein 4, *LCI* lipoprotein combine index

## Discussion

In this cross-sectional study, we discovered that a combination of the RBP-4 level and the LCI value can be used to diagnose ACS and found that RBP-4 + LCI was a novel independent risk factor for ACS. The main findings of this study are as follows: First, per ROC curve analysis, the AUC of the LCI + RBP-4 group was higher than the AUC of the individual LCI and RBP-4 groups, and this indicated that the accuracy of ACS diagnosis using combined RBP-4 + LCI values was higher than the accuracy of RBP-4 or LCI alone. Second, RBP-4 ≥4 ng/ml, LCI ≥16 and LCI ≥16 + RBP-4 ≥4 ng/ml were all new independent risk factors of ACS besides the classical risk factors of CAD. Interestingly, the risk associated with LCI ≥16 + RBP-4 ≥4 ng/ml was higher than the risk of LCI ≥16 or RBP-4 ≥4 ng/ml alone. Third, the risk of RBP-4 ≥4 ng/ml for the occurrence of lesion 2 vessels is highest, while the risk of LCI ≥16 for the occurrence of 1 lesion vessel is highest. There is growing evidence supporting that the presence and severity of the cardiovascular disease can be detected using certain biomarkers and components of the lipid profile. However, to our knowledge, this is the first study that has reported the association among LCI ≥16, RBP and ACS [14–16].

There is increasing data strongly supporting the fact that the serum level of RBP-4 is associated with cardiovascular disease as well as adverse cardiovascular outcomes [17, 18]. Liu et al. [19] conducted a cross-sectional and prospective study, which reported that the level of RBP-4 is significantly associated with the complexity of coronary lesion in patients with ACS. A cross-sectional study by Lambadiari et al. [20] found that RBP-4 is a strong predictor of CAD, and independently correlated its levels with CAD severity. Both these conclusions are consistent with our results. Additionally, our findings also extend the reports of these previous studies by showing that the level of RBP-4 showed an upward trend with the increase in the number of lesions in the vessels, and the risk of RBP-4 ≥4 ng/ml for occurrence of 2 vessels lesion was the highest compared to 1 or ≥3 vessels lesion group. Recent studies have demonstrated that RBP-4 contributes to inflammatory injury of cardiomyocytes through Toll-like receptor 4 activation and myeloid differentiation through the primary response gene 88 (MyD88) pathway [21]. Additionally, pathophysiological mechanism of insulin resistance, type 2 diabetes, and endothelial dysfunction occurs through extracellular signal-regulated kinase activity [7, 22, 23].

The patients with ACS had higher levels of TC, TG, LDL-C and lower level of HDL-C than those in the control group in previous studies [24, 25]. There is evidence indicating that TC, TG, LDL-C and HDL-C levels could be used as risk markers of cardiovascular disease with a strong correlation with coronary atherosclerosis [16, 26, 27]. However, the HDL-C levels had no statistical difference between the two groups in our study.TG-rich lipoproteins, TG, and LDL-C play roles in promoting inflammation, thrombosis, and insulin resistance, which are related to the formation, development and vulnerability of coronary plaque [28–30]. In addition to participating in reverse cholesterol transport, HDL-C also has antioxidant, anti-inflammatory, and anti-atherosclerotic effect [31]. Previous studies have shown that the ratios of TG/HDL-C, TC/HDL-C, LDL-C/HDL-C are significantly associated with insulin resistance [32]. Further, the ratio of TG/HDL-C is related to the severity of CAD and can be used as a significant predictor of ACS in young adults, while non-HDL-C/TC is a new marker to assess the severity of coronary artery lesions and cardiovascular outcomes [26, 33]. Although Wu et al. [10] proposed the importance of LCI value, they did not report LCI as a significant risk predictor of CAD. However, our study has clearly shown that LCI can be used as a risk factor for ACS, and the risk associated with it was similar to the risk of hypertension and diabetes mellitus.

In the past few decades, a large number of studies have been conducted to understand the pathogenesis, clinical prevention and treatment of cardiovascular diseases. Studies have also confirmed that adipose tissues such as epicardial adipose tissue, pericardial adipose tissue, and thoracic adipose tissue secrete adipocytokines such as adiponectin, tumor necrosis factor-α (TNF-α), and omentin-1. Moreover, the clinical risk factors such as diabetes, hypertension, dyslipidemia are strongly linked with the pathophysiological mechanisms of CAD [14, 34–38]. Although these efforts have improved the screening, diagnosis, treatment, and prognosis of CAD, the prevalence of CAD remains high and it is still one of the major health issues in the world. ACS remains to be the most common cause of death worldwide, and the mortality due to ACS continues to rise [39]. Previous studies have focused on the unilateral effects of adipocytokines or clinical risk factors or adipose tissue on CAD. In this study, we have shown that when the levels adipocytokines such as RBP-4 were combined with clinical markers related to the diagnosis of ACS, the diagnostic accuracy of the combined indicator was higher than that of the single indicator, and the risk associated with the combined indicator was higher than the single indicator. In addition, we have also found that the numbers of stenotic vessels increased with increase in risk factors such as risk of hypertension, diabetes mellitus, smoking and history of MI. These findings might help in further promoting the diagnosis, prevention, and treatment of ACS.

In this study, the risk of LCI ≥16 for the occurrence of 1 lesion vessel was surprisingly higher than the risk of occurrence of 2 or ≥3 lesion vessels. We searched Medline by “lipoprotein combine index” and got three literatures only. A cross- sectional study found that LCI correlated with the atherosclerotic vascular disease better than serum lipid parameters [40]. Stephen et al. found that LCI was a predictive indicator of CAD [10, 41]. However, no literatures above were correlated with LCI and the numbers of stenotic coronary arteries. We are so sorry that the true mechanisms of LCI in coronary artery lesions is still unclear. Therefore, further researches are needed to tell the whole story then.

This study had some limitations. First, the sample size is relatively small, and as a result, there might be a selection bias. Second, as this is a single-center study, all inpatients were enrolled from one hospital within a specific period of time. Hence, a multi-regional and multi-ethnic study with a large sample size is needed in the future. Third, our study only explored the combined effects of RBP-4 and LCI on ACS, and the role of other adipocytokines and clinical risk factors along with these factors need to be further explored.

In conclusion, increase of the RBP-4 and LCI values were found to be independent risk factors for ACS, and the risk associated with the combined rise of LCI and RBP-4 was higher than the risk of LCI or RBP-4 alone. Therefore, combined tests assessing the LCI and RBP-4 values might be a potential diagnostic marker for ACS. However, as a single-center pilot study with a small sample size, the conclusions may have some limitations indeed. Our team will do more research work on ACS territory in the future clinical practice.
